# Supplementary material for: Usage frequency and lexical class determine the evolution of kinship terms in Indo-European
Source: R Soc Open Sci. 2019 Oct 30;6(10):191385. doi: 10.1098/rsos.191385 (PMC6837234; doi:10.1098/rsos.191385)
Supplement: Supplementary Material [file rsos191385supp1.pdf]

Supplementary Information, Usage frequency and lexical class determine the evolution of kinship terms in Indo-European

Rácz, Passmore, Sheard, and Jordan

Contents

Kinship data **1**
Supplementary data table . . . 2
Data summary . . . 3
Frequency data . . . 3

Cognate data **7**
Phylogeny . . . 7
Rates of change . . . 7
Example BayesTraits script . . . 10
Half-life . . . 10

Frequency of use and rates of change: Swadesh words and kin terms **11**
Control model: kin terms . . . 11
Predictive model: rate of change and frequency of use . . . 12

References **14**

Kinship data

We collected kin terms from 45 languages for the following relations: B, D, F, M, MB, MZ, MZD, MZS, S, Z (i.e. brother, daughter, father, mother, mother's brother, mother's sister, mother's sister's daughter, mother's sister's son, son, sister), collected from a combination of native speakers, ethnographies, and dictionaries in our 'Kinbank' database. Initially, the analyses used a broader set of kinship terms (e.g. FB, FZ, BW, ZH, i.e. father's brother, father's sister, brother's wife, sister's husband), however, we restricted the sample to form a comparable set of word frequencies. This is because most Indo-European languages do not have separate terms for e.g. MZD and MBD and separate terms for e.g. BW are exceedingly rare (both across languages as types and within languages as tokens). We have not included *husband* and *wife*, because these are often synonymous with *man* and *woman*, respectively.

Frequency data were collected from 34 corpora in 21 languages in three corpus types: spoken, written, and web-crawled. The list of corpora is in Table S1 below.

Table S1: Source of frequency data (source of words is Kinbank)

Language	Corpus name	Reference
Albanian	Albanian NC	(Morozova 2015)
Bulgarian	Bulgarian NC	(Koeva, Blagoeva, and Kolkovska 2010)
Bulgarian	Bulgarian Web 2012 (bgTenTen12)	(Kilgarriff et al. 2014)
Catalan	Catalan Web 2014 (caTenTen14)	(Kilgarriff et al. 2014)
English	CELEX	(Baayen, Piepenbrock, and H 1993)
German	CELEX	(Baayen, Piepenbrock, and H 1993)
Spanish	CoE	(Moreno-Sandoval et al. 2005)

Language	Corpus name	Reference
Portuguese	CoP	(Craveiro, Macedo, and Madeira 2012)
Italian	CORIS	(Favretti, Tamburini, and De Santis 2002)
Czech	Czech Web 2012 (czTenTen12 v8)	(Kilgarriff et al. 2014)
Danish	Danish Web 2014 (daTenTen14)	(Kilgarriff et al. 2014)
Dutch	DINL (written) CGN (spoken)	(Kruyt and others 1997)
Dutch	DINL (written) CGN (spoken)	(Van Eerten 2007)
Dutch	Dutch Web 2014 (nlTenTen14)	(Kilgarriff et al. 2014)
French	French Web 2012 (frTenTen12)	(Kilgarriff et al. 2014)
German	German Web 2013 (deTenTen13)	(Kilgarriff et al. 2014)
Greek	Greek Web 2014 (elTenTen14)	(Kilgarriff et al. 2014)
Croatian	hrWaC	(Kilgarriff et al. 2014)
Italian	Italian Web 2010 (itTenTen)	(Kilgarriff et al. 2014)
French	lexique corpus	(New et al. 2001)
Norwegian	Norwegian Web 2015 (noTenTen15)	(Kilgarriff et al. 2014)
Polish	Polish NC written PELCRA spoken	(Przepiórkowski et al. 2008)
Polish	Polish NC written PELCRA spoken	(Przepiórkowski et al. 2010)
Polish	Polish Web 2012 (plTenTen12)	(Kilgarriff et al. 2014)
Portuguese	Portuguese Web 2011 (ptTenTen11, Freeling v3)	(Kilgarriff et al. 2014)
English	pukWaC (ukWaC parsed with MaltParser)	(Kilgarriff et al. 2014)
Romanian	Romanian Web (roWaC)	(Kilgarriff et al. 2014)
Romanian	ROMBAC	(Ion et al. 2012)
Russian	Russian NC via Leeds	(Lyashevskaya and Sharov 2009)
Russian	Russian Web 2011 (ruTenTen11)	(Kilgarriff et al. 2014)
Serbian	scWaC	(Kilgarriff et al. 2014)
Spanish	Spanish Web 2011 (esTenTen11, Eu + Am, Freeling v4)	(Kilgarriff et al. 2014)
Swedish	SUC	(Gustafson-Capková and Hartmann 2006)
Swedish	Swedish Web 2014 (svTenTen14)	(Kilgarriff et al. 2014)
Icelandic	textasafn	(Úlfarsdóttir and Bjarnadóttir 2017)
Czech	UCNK:SYN2015 (written) ORAL2013 (spoken)	(Čermák 1997)
Czech	UCNK:SYN2015 (written) ORAL2013 (spoken)	(Benešová, Kren, and Waclawicová 2013)

The analysis uses one term per language per kinterm. If it arises that a language has multiple words for a particular kinterm, we use which ever was more frequent in the corpus data. In the case where a language has multiple words for a single kinterm, and we do not have access to frequency data, we rely on expert judgement to select a term.

Supplementary data table

See the supplementary data csv file for the raw data used in this study.

Column description:

Table S2: Supplementary table column descriptions

Column name	Description
meaning	Code used for kinterm, see TS1 for a description
language	Common name for each language
word	Words used in the analysis
lingpy.cognate	Cognates as determined by lingpy, used as a first pass
expert.cognate	Cognates reviewed and corrected by expert reviewers, used in analysis

Column name	Description
word.count	Frequency of each term in the given corpus
corpora.size	Total size of the corpus
corpora.type	Type of corpus used (either web, written, or spoken)
corpus	Name of the corpus
states	States
taxa	Taxa label in phylogeny
glottocode	Glottocode
source.ids	Standard deviation for the global rate of replacement
mean.roc	Mean global rate of replacement
sd.roc	Source of term (see source SM)

Data summary

We estimate rate of replacement and compare it with frequency of use for ten types of kin relations. Here we use MB, MZ, MZS, and MZD as shorthand for broader terms (uncle, aunt, male cousin, and female cousin, respectively). This is because, as we note in the first section, commonly used terms in this set of languages do not distinguish terms according to the parent's gender.

Below is a table indicating the shorthand used for each kinterm, the number of languages for which we have data, and the number of states (or cognates) for that term.

We collected terms for a superset of the languages from which frequency data is available in order to have a more robust estimate of rate of replacement for each term.

Table S3: Each kinterm used, with counts for the number of languages we have data for and the number of cognates across our sample

Kinterm	Description	No. languages	States
F	Father	45	7
M	Mother	45	5
S	Son	43	7
D	Daughter	43	10
B	Brother	45	9
Z	Sister	44	6
MB	Mother's brother (uncle)	39	7
MZ	Mother's sister (aunt)	39	10
MZS	Mother's sister's daughter (female cousin)	31	11
MZD	Mother's sisters son (male cousin)	31	11

Frequency data

Below are bar graphs of term frequency by language, across corpora types (web, written, or spoken).

Figure S1: Web frequencies of terms across languages

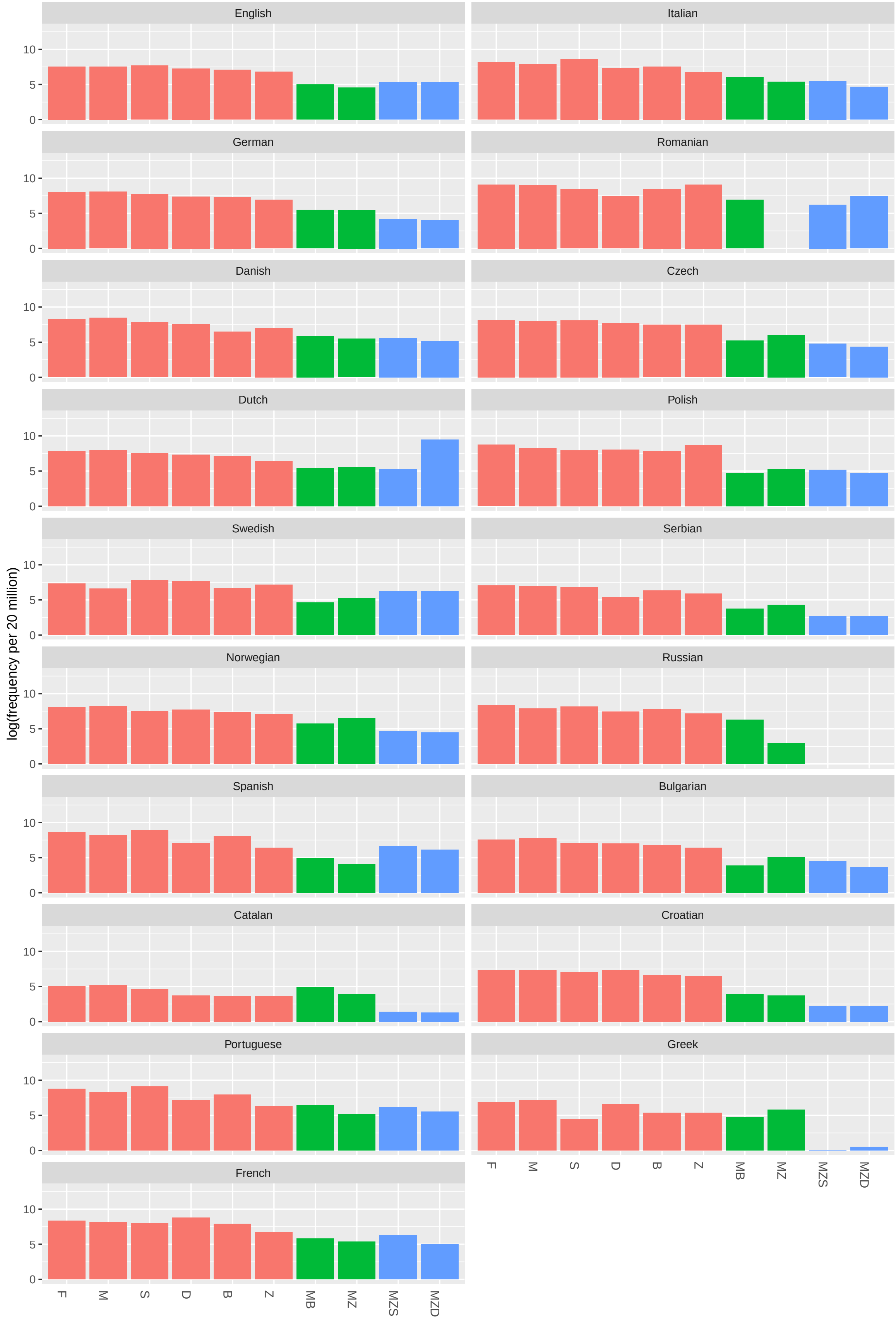

Figure S2: Written frequencies of terms across languages

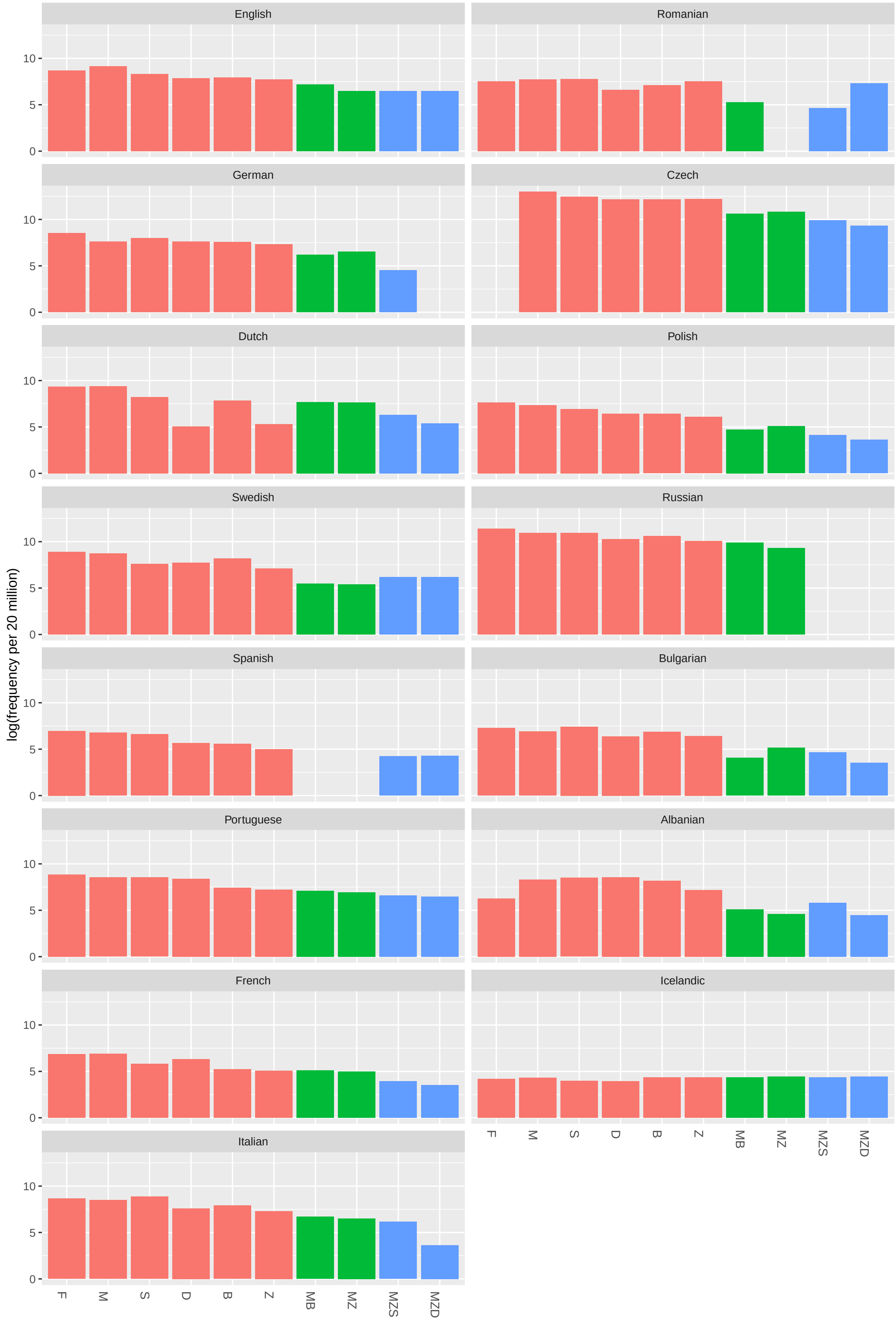

Figure S3: Spoken frequencies of terms across languages

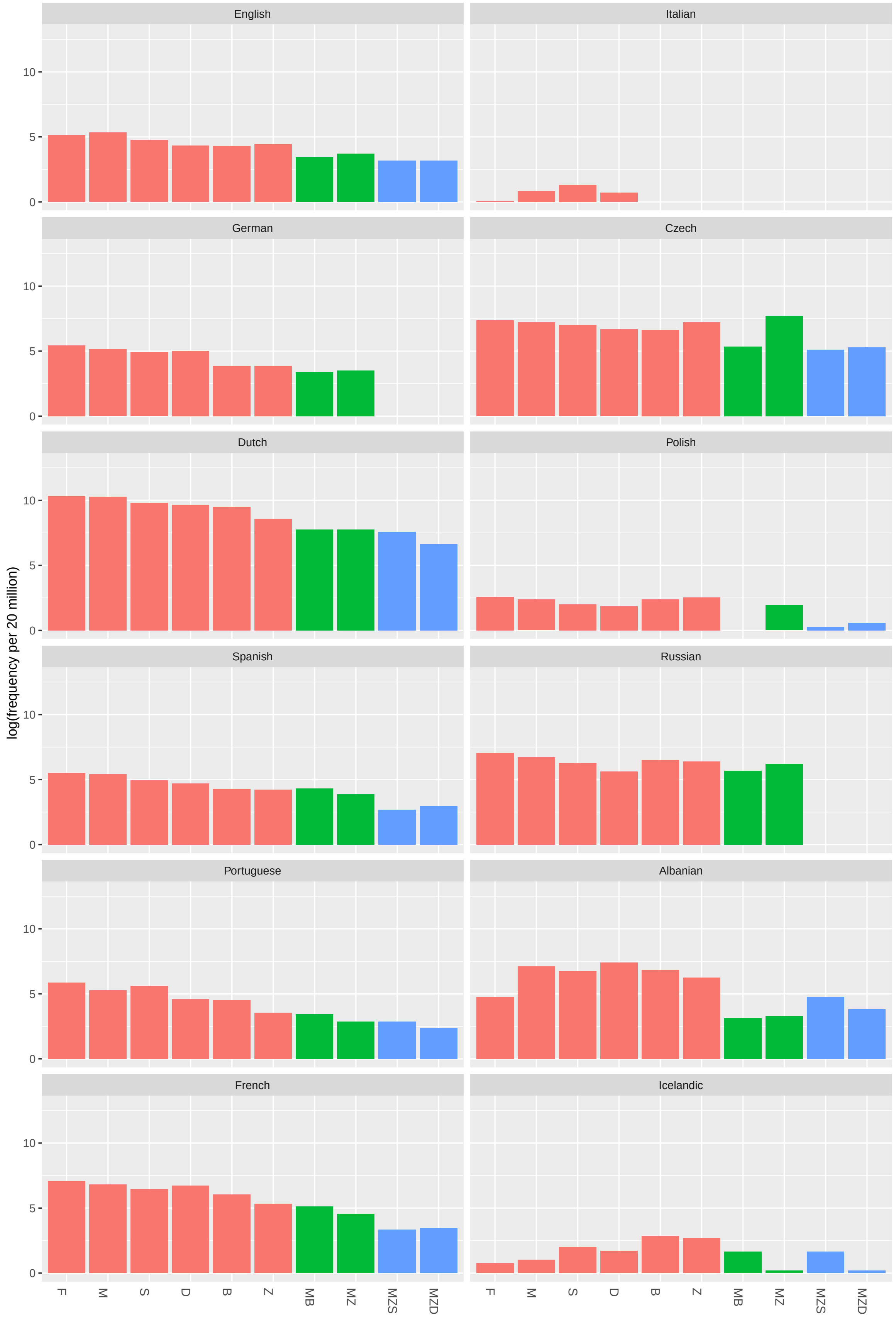

Cognate data

We generated cognate classes using the Indo-European Etymological Dictionary (Buck 2008), LingPy (List, Greenhill, and Forkel 2018), and a panel of volunteer experts, recruited on Linguist List (all faults remain ours). All terms were automatically transcribed into the Speech Assessment Methods Phonetic Alphabet (SAMPA) through LingPy's `uni2sampa` function. Cognates were automatically allocated using LingPy's `cluster` function. Using the cognate-coded Swadesh list, the list of core vocabulary terms (subset to those languages for which we also have kinterms), we tested the appropriateness of edit distance, SCA, and turchin algorithms, alongside Phonemic and Phonetic transcriptions for cognate detection in our data, following the code examples from Lingpy.org. The F-score was highest for the edit-distance algorithm with a 0.4 threshold (see table S4) (List, Greenhill, and Forkel 2018). We then manually adjusted the results, followed by expert review which resulted in minor changes. Automatic decisions and the corrections are available in the supplementary data file.

Table S4: Precision, recall, & F-score for various LingPy cognate detection algorithms and their threshold settings.

transcription	threshold	method	precision	recall	fscore
Phonemic	0.4	edit	0.7792	0.4238	0.5490
Phonemic	0.6	edit	0.6259	0.4247	0.5060
Phonemic	0.4	turchin	0.5152	0.4271	0.4670
Phonemic	0.6	turchin	0.5152	0.4271	0.4670
Phonetic	0.4	edit	0.8434	0.3194	0.4634
Phonemic	0.4	sca	0.4821	0.4273	0.4530
Phonemic	0.6	edit	0.6944	0.3204	0.4385
Romanised	0.4	edit	0.6930	0.2948	0.4136
Phonetic	0.4	turchin	0.4524	0.3213	0.3758
Phonetic	0.6	turchin	0.4524	0.3213	0.3758
Phonetic	0.6	turchin	0.4524	0.3213	0.3758
Romanised	0.6	edit	0.5079	0.2958	0.3739
Phonetic	0.4	sca	0.4418	0.3221	0.3726
Romanised	0.4	sca	0.4491	0.2962	0.3570
phoneMic	0.6	sca	0.3011	0.4294	0.3539
Romanised	0.4	turchin	0.4290	0.2963	0.3505
Romanised	0.6	turchin	0.4290	0.2963	0.3505
Phonetic	0.6	sca	0.2262	0.3244	0.2666
Romanised	0.6	sca	0.2227	0.2983	0.2550

Phylogeny

We used 1000 phylogenies from the most recent Bayesian posterior of Indo-European phylogenies (Bouckaert et al. 2012). Trees in the sample are rooted. Branch lengths are given in years and derived from statistical and historical calibration. The Indo-European posterior used has an approximate age of 8,700 years. Trees initially have 111 taxa, and these were pruned down for each kinterm dependent on available data. Counts for taxa for each kinterm can be found in table S3. By using a sample of likely phyloygenies and through using a Bayesian approach, we account for the phylogenetic uncertainty.

Rates of change

Table S3 shows the number of languages and states used to estimate rate of change for each kinterm. Each language is linked to a taxon in the Indo-European phylogeny. Following the methods in Pagel and Meade

(2018) we use BayesTraits version 3.0.1 to implement a Bayesian MCMC approach to estimate the instantaneous global rate of change for each kin-term through Q-matrix normalisation. Probabilities of frequency were scaled to represent the empirical frequencies. We used a stepping-stone sampler, using 100 stones for 1000 iterations each. MCMC chains ran for a total of 10,010,000 iterations, with a burn-in of 10,000, sampling every 1000 iterations. This left a posterior sample of 10,000 iterations, which is approximately 10 samples per tree. To make the rates comparable to Pagel et al., we scale instantaneous rates to change per 10,000 years.

Each analysis was run 3 times to ensure the MCMC chain converged. Tables S5 - S14 display the marginal log-likelihood for each MCMC run, the mean global rate of change for each run, and the average across the three runs. Each table is labelled by kin code. For each kinterm, we also used the Gelman-Rubin diagnostic test for convergence (Gelman and Rubin 1992). This tests for MCMC convergence between multiple chains by analysing the differences between them. By estimating a 'potential scale reduction factor', which when multiplying across chains would remove the differences, we can quantify the differences between chains (a scale reduction factor 1 indicating no change needed). A rule of thumb suggests a point estimates of less than 1.1 is sufficient to claim convergence, and ensuring upper limits are also around these limits.

Table S5: B

	Marginal log-likelihood	Harmonic Mean global rate	Harmonic SD global rate
1	50.38400	0.0001267	5e-07
2	47.36400	0.0001240	4e-07
3	49.74900	0.0001219	4e-07
Mean	49.16567	0.0001242	4e-07

Table S6: D

	Marginal log-likelihood	Harmonic Mean global rate	Harmonic SD global rate
1	67.15900	0.0002732	8e-07
2	66.91900	0.0002718	8e-07
3	70.64700	0.0002704	9e-07
Mean	68.24167	0.0002718	8e-07

Table S7: F

	Marginal log-likelihood	Harmonic Mean global rate	Harmonic SD global rate
1	69.12500	0.0002581	8e-07
2	71.08600	0.0002657	9e-07
3	65.72500	0.0002615	9e-07
Mean	68.64533	0.0002618	8e-07

Table S8: M

	Marginal log-likelihood	Harmonic Mean global rate	Harmonic SD global rate
1	57.339	0.0002995	1e-06
2	57.493	0.0002999	9e-07
3	59.432	0.0002995	1e-06
Mean	58.088	0.0002996	1e-06

Table S9: MB

	Marginal log-likelihood	Harmonic Mean global rate	Harmonic SD global rate
1	57.44400	0.0003308	1.1e-06
2	57.04900	0.0003303	1.2e-06
3	57.78000	0.0003401	1.1e-06
Mean	57.42433	0.0003337	1.1e-06

Table S10: MZ

	Marginal log-likelihood	Harmonic Mean global rate	Harmonic SD global rate
1	74.88300	0.0005056	1.8e-06
2	73.92900	0.0005090	1.8e-06
3	68.98400	0.0005106	1.7e-06
Mean	72.59867	0.0005084	1.7e-06

Table S11: MZD

	Marginal log-likelihood	Harmonic Mean global rate	Harmonic SD global rate
1	63.392	0.0003877	1.3e-06
2	58.497	0.0003839	1.4e-06
3	57.940	0.0003916	1.3e-06
Mean	59.943	0.0003877	1.4e-06

Table S12: MZS

	Marginal log-likelihood	Harmonic Mean global rate	Harmonic SD global rate
1	61.62400	0.0004162	1.3e-06
2	62.33400	0.0004158	1.3e-06
3	60.83500	0.0004151	1.4e-06
Mean	61.59767	0.0004157	1.4e-06

Table S13: S

	Marginal log-likelihood	Harmonic Mean global rate	Harmonic SD global rate
1	47.75800	0.0001669	7e-07
2	50.56400	0.0001666	7e-07
3	48.46100	0.0001711	6e-07
Mean	48.92767	0.0001682	7e-07

Table S14: Z

	Marginal log-likelihood	Harmonic Mean global rate	Harmonic SD global rate
1	42.31300	0.0001205	5e-07
2	44.06800	0.0001215	6e-07

	Marginal log-likelihood	Harmonic Mean global rate	Harmonic SD global rate
3	44.12300	0.0001265	5e-07
Mean	43.50133	0.0001229	5e-07

Table S15: Point estimates and upper 95% confidence limits for Gelman-Rubin MCMC diagnostic tests

	Point est.	Upper C.I.
Table S5: B	1.00	1.01
Table S6: D	1.00	1.00
Table S7: F	1.00	1.01
Table S8: M	1.00	1.00
Table S9: MB	1.00	1.00
Table S10: MZ	1.01	1.02
Table S11: MZD	1.00	1.02
Table S12: MZS	1.00	1.00
Table S13: S	1.00	1.00
Table S14: Z	1.00	1.00

Example BayesTraits script

```
BayesTraitsV3 tree.file data.file
1
2
NQM
Pis Emp
RevJump exp 10
Stones 100 1000
Iterations 10010000
Burnin 10000
Sample 10000
LogFile logs/file
run
```

Half-life

We calculate the half-life of each kinterm following methods from Pagel and Meade (2018). The half-life of a term estimates the expected amount of time before a 50% chance of a cognate change.

Table S16: Mean half-life for each kin code

Kin code	Half-life (years)
B	5471
D	2537
F	2686
M	2315
MB	2096
MZ	1371
MZD	1788
MZS	1666

Kin code	Half-life (years)
S	4152
Z	5751

Frequency of use and rates of change: Swadesh words and kin terms

We want to see if

- Rate of change correlates with frequency of use for kin terms
- What the strength of this relationship is compared to Swadesh terms

The difficulty is that the two data sets are structured differently. A given **kin term** can have a written / spoken / web frequency as well as a word / lemma frequency. A given **Swadesh term** (core vocabulary term) only has one frequency (though it may be written / spoken / etc. depending on the source corpus). Term **length** correlates with frequency of use in a way that is not directly relevant to our analysis either.

In order to create comparable kin- and Swadesh-datasets, we fit a linear mixed model (M1.1) as control on the kinterm data and use word meaning random intercepts from this model in a second, predictive, model (M2)

Control model: kin terms

Model 1.1:

Centralised log frequency of use per million ~ Corpus genre + Frequency type + (1 | Word meaning)

The aim of M1.1 is to provide us with a word meaning random intercept for M, F, B, Z, etc. that incorporates genre and frequency type information. As a result, random slopes were not tested. Table S17 shows the fixed effects for this model.

The word meaning-level random intercepts capture word frequency across data sources. The intercepts predict rate of change, even when controlling for word length.

Table S17: Summary of fixed effects for control model M1.1

	Estimate	Std. Error	t value
(Intercept)	-0.54	0.21	-2.56
genreweb	1.68	0.17	10.03
genrewritten	2.61	0.18	14.58
freq.typeword	-0.74	0.13	-5.50

Model 1.2:

Centralised rate of replacement ~ Frequency measure + Word length + (1 | Language)

Since the frequency measure and word length are both word-level predictors, we have no potential random slopes and report the model with a random intercept for language only. The estimates of the fixed effects can be seen in table S18.

M1.1 provides us with aggregated information on the centralised log frequency per million of each kinship word. This allows direct comparison with the core vocabulary (where we only have one datum per word) without a considerable loss of information.

M1.2 only serves to demonstrate that the frequency effect is not an artefact of word length.

Table S18: Summary of fixed effects for control M1.2

	Estimate	Std. Error	t value
(Intercept)	0.64	0.31	2.03
frequency.measure	-0.58	0.07	-8.72
word.length	-0.10	0.06	-1.76

Predictive model: rate of change and frequency of use

Model 2.1:

Centralised rate of replacement ~ Frequency measure * Word type + (1 | Language)

Model 2.2:

Centralised rate of replacement ~ Frequency measure * Word type + (Word type | Language)

The predictive model (M2) uses the random word-meaning intercepts (named Frequency measure) from M1.1 as measures of frequency of use for kin terms, and centralised log frequency per million for the Swadesh terms. We restrict the dataset to languages for which we have kin term data. We propose two possible random effect structures, either random intercepts for each language (M2.1), or random slopes for each word type in each language (M2.2. Word-type is the only fixed effect that can vary across language. Goodness-of-fit tests reveal that this random slope results in a better fit (Table S19), so we report M2.2, the model with the slope (Table S20). The interaction effect is plotted in figure S5. Figure S6 shows a plot of the raw data used in the model, highlighting the kinship terms.

Table S19: Goodness for fit for model random effect structures

term	df	AIC	BIC	logLik	deviance
M2.1	6	7770.45	7804.02	-3879.23	7758.45
M2.2	8	7768.48	7813.24	-3876.24	7752.48

Table S20: Summary of fixed effects for predictive model M2.2

	Estimate	Std. Error	t value
(Intercept)	0.12	0.12	1.01
frequency.measure	-0.44	0.08	-5.26
word.typeswadesh.word	-0.54	0.18	-2.91
frequency.measure:word.typeswadesh.word	0.18	0.09	2.02

Figure S5: M2.2 rate of change ~ freq : word type

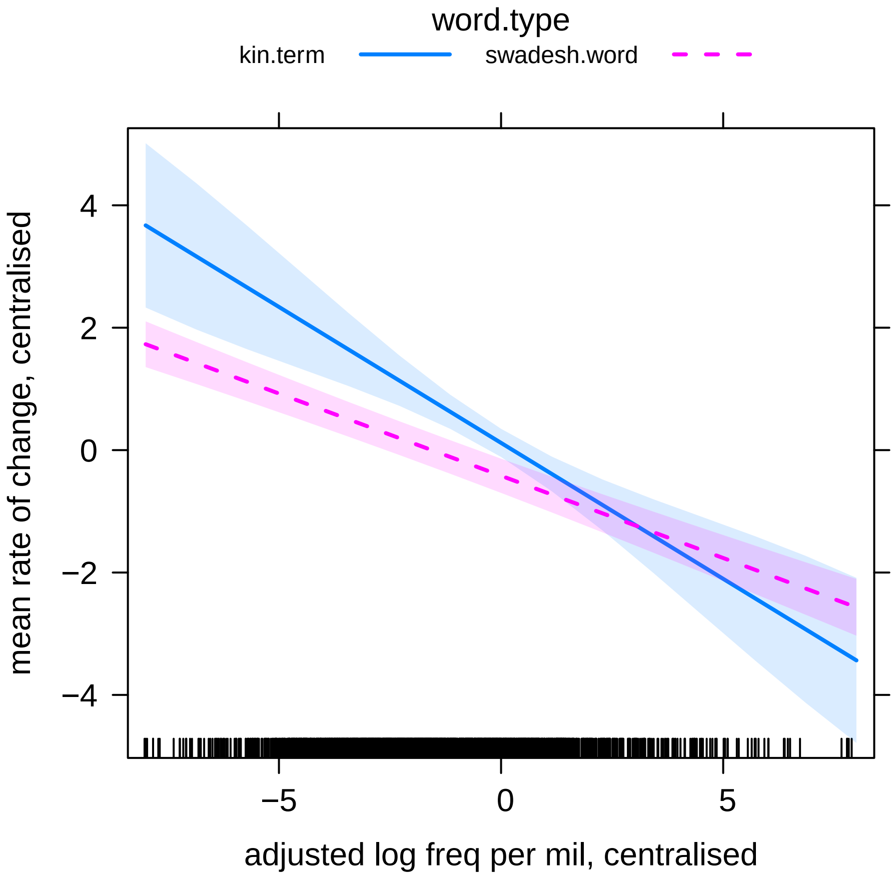

Figure S6: Mean raw data correlation of rate of change and frequency measure in predictive model

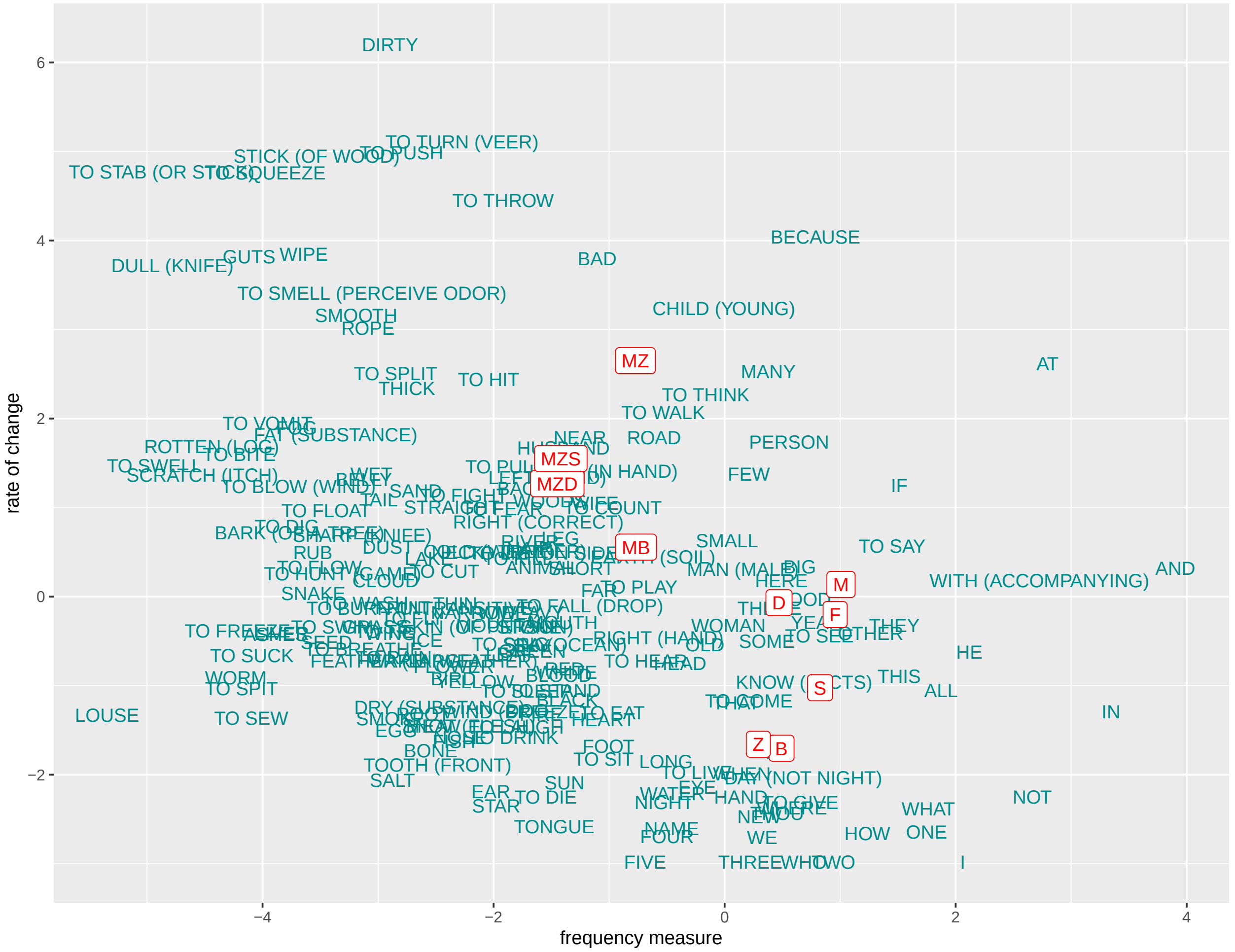

References

Baayen, R Harald, Richard Piepenbrock, and Rijn van H. 1993. "The {Celex} Lexical Data Base on {Cd-Rom}." Linguistic Data Consortium.

Benešová, L, M Kren, and M Waclawicová. 2013. "ORAL2013: Reprezentativni Korpus Neformálni Mluvené Češtiny [Oral2013: A Representative Corpus of Informal Spoken Czech]." *Ustav ˇCeského Národniho Korpusu FF UK, Praha.*

Bouckaert, Remco, Philippe Lemey, Michael Dunn, Simon J. Greenhill, Alexander V. Alekseyenko, Alexei J. Drummond, Russell D. Gray, Marc A. Suchard, and Quentin D. Atkinson. 2012. "Mapping the Origins and Expansion of the Indo-European Language Family." *Science* 337 (6097): 957–60. doi:10.1126/science.1219669.

Buck, Carl Darling. 2008. *A Dictionary of Selected Synonyms in the Principal Indo-European Languages*. University of Chicago Press.

Craveiro, Olga, Joaquim Macedo, and Henrique Madeira. 2012. "It Is the Time for Portuguese Texts!" In *International Conference on Computational Processing of the Portuguese Language*, 106–12. Springer.

Čermák, Franti ek. 1997. "Czech National Corpus: A Case in Many Contexts." *International Journal of Corpus Linguistics* 2 (2). John Benjamins Publishing Company: 181–97.

Favretti, R Rossini, Fabio Tamburini, and Cristiana De Santis. 2002. "CORIS/Codis: A Corpus of Written

Italian Based on a Defined and a Dynamic Model." *A Rainbow of Corpora: Corpus Linguistics and the Languages of the World*, 27–38.

Gelman, Andrew, and Donald B Rubin. 1992. "Inference from Iterative Simulation Using Multiple Sequences." *Statistical Science*. JSTOR, 457–72.

Gustafson-Capková, Sofia, and Britt Hartmann. 2006. "Manual of the Stockholm Umeå Corpus Version 2.0." *Stockholm University*.

Ion, Radu, Elena Irimia, Dan Stefanescu, and Dan Tufis. 2012. "ROMBAC: The Romanian Balanced Annotated Corpus." In *LREC*, 339–44. Citeseer.

Kilgarriff, Adam, Vít Baisa, Jan Bušta, Miloš Jakubíček, Vojtěch Kovář, Jan Michelfeit, Pavel Rychly, and Vít Suchomel. 2014. "The Sketch Engine: Ten Years on." *Lexicography* 1 (1). Springer: 7–36.

Koeva, Svetla, Diana Blagoeva, and Siya Kolkovska. 2010. "Bulgarian National Corpus Project." *Politics* 207: 2–2.

Kruyt, MWF, and others. 1997. "A 38 Million Words Dutch Text Corpus and Its Users." *Lexikos* 7 (7). Bureau of the WAT: 229–44.

List, Johann-Mattis, Simon J. Greenhill, and Robert Forkel. 2018. "LingPy. A Python Library for Historical Linguistics." http://lingpy.org.

Lyashevskaya, ON, and SA Sharov. 2009. "Chastotnyj Slovar'sovremennogo Russkogo Iazyka (Na Materialakh Natsionalnogo Korpusa Russkogo Iazyka)[Frequency Dictionary of Modern Russian Based on the Russian National Corpus]." *Moscow: Azbukovnik*.

Moreno-Sandoval, Antonio, Gillermo De la Madrid, Manuel Alcántara, Ana Gonzalez, José Maria Guirao, and Raúl De la Torre. 2005. "The Spanish Corpus." *C-ORAL-ROM: Integrated Reference Corpora for Spoken Romance Languages, Amsterdam: John Benjamins Publishing Company*, 135–61.

Morozova , Rusakov . 2015. "Albanian National Corpus: Composition, Text Processing and Corpus-Oriented Grammar Development." *Sprache Und Kultur Der Albaner. Zeitliche Und Räumliche Dimensionen.* 5: 270–308.

New, Boris, Christophe Pallier, Ludovic Ferrand, and Rafael Matos. 2001. "Une Base de Données Lexicales Du Français Contemporain Sur Internet: LEXIQUE™//a Lexical Database for Contemporary French: LEXIQUE™." *L'année Psychologique* 101 (3). Persée-Portail des revues scientifiques en SHS: 447–62.

Pagel, Mark, and Andrew Meade. 2018. "The Deep History of the Number Words." *Phil. Trans. R. Soc. B* 373 (1740): 20160517. doi:10.1098/rstb.2016.0517.

Przepiórkowski, Adam, Rafal L Górski, Marek Lazinski, and Piotr Pezik. 2010. "Recent Developments in the National Corpus of Polish." In *LREC*.

Przepiórkowski, Adam, Rafal L Górski, Barbara Lewandowska-Tomaszyk, and Marek Lazinski. 2008. "Towards the National Corpus of Polish." In *LREC*.

Úlfarsdóttir, Thórdís, and Kristín Bjarnadóttir. 2017. "The Lexicography of Icelandic." *International Handbook of Modern Lexis and Lexicography*. Springer, 1–12.

Van Eerten, Laura. 2007. "Over Het Corpus Gesproken Nederlands." *Nederlandse Taalkunde* 12 (3): 194–215.
